# Supplementary material for: Development and validation of artificial intelligence to detect and diagnose liver lesions from ultrasound images
Source: PLoS One. 2021 Jun 8;16(6):e0252882. doi: 10.1371/journal.pone.0252882 (PMC8186767; doi:10.1371/journal.pone.0252882)
Supplement: S1 File — (PDF) [file pone.0252882.s001.pdf]

S1 File

Table of contents

	Page
**Supporting Appendices**	
- **S1 Appendix.** Image preprocessing	2
- **S2 Appendix.** Intersection-over-Union (IoU)	3
- **S3 Appendix.** Selection of confidence threshold	4
- **S4 Appendix.** Proof of independence between confidence threshold and classification performance	5
**Supporting Figures**	
- **S1 Fig.** Intersection-over-Union (IoU)	6
- **S2 Fig.** Examples of detected FLLs with different IoUs	7
- **S3 Fig.** Histogram shows distribution of IoUs of detected FLLs	8
- **S4 Fig.** Examples of normal organs and lesions in other organs falsely detected as FLLs	9
- **S5 Fig.** Examples of false-negatively detected images	10
- **S6 Fig.** Examples of misclassified FLLs	11
- **S7 Fig.** Examples of images before and after markers were removed	12
- **S8 Fig.** Plots between score thresholds versus recalls, precisions and F2 scores for detecting each of the 5 FLL diagnoses	13
- **S9 Fig.** Plots between score thresholds versus overall detection rates, diagnostic sensitivities and specificities	16
**Supporting Tables**	
- **S1 Table.** Ultrasound machine models from which images were taken	17
- **S2 Table.** False-positive detection of other structures as FLLs	18
- **S3 Table.** Causes of false negative detections	20
- **S4 Table.** Causes of misclassifications	21
- **S5 Table.** Subgroup analysis by FLL sizes	22
- **S6 Table.** Subgroup analysis by background liver parenchyma	23
- **S7 Table.** Top-1, top-2 and top-3 diagnostic sensitivities and specificities for HCC	24
- **S8 Table.** Training hyperparameters for RetinaNet	25

S1 Appendix. Image preprocessing

During image preprocessing, all patient identification information and the peripheral areas in the USG images were cropped out. We identified the coordinates of fan-shaped USG region by 'Sequence of Ultrasound Regions' DICOM header, in order to ensure that the cropped image contained only the fan-shaped USG region where annotations and dimension measurements had been cropped out. The images were then resized to 1333 pixels wide and 800 pixels tall before inputted into the CNN. Since USG images were retrospectively retrieved from the PACS system, some images contained markers made by sonographers. We developed another AI system based on a framework called Generative Adversarial Network (GAN) to remove the markers [1]. To train GAN, a small region within each image without marker was randomly removed. Then, GAN was trained to restore the removed region by using the surrounding context as clue. After GAN training was completed, regions containing markers were identified and removed. Then, the trained GAN restored the removed regions, i.e. the restored regions no longer had markers. This process was done in order to prevent the CNN to learn to detect the lesion by recognizing the markers instead of FLLs' features. Examples of images before and after marker removal are illustrated in **S7 Fig**.

S2 Appendix. Intersection-over-Union (IoU)

Intersection-over-Union (IoU) was used to evaluate the matching area between a predicted bounding box and a bounding box around the area of the true location.

$$Intersection\ over\ Union\ (IoU) = \frac{|G \cap P|}{|G \cup P|}$$

where G and P are the set of pixels belonging to the bounding box of the true location and the predicted bounding box, respectively (**S1 Fig**). A predicted bounding box was counted as correct detection if its location matched the bounding box of the true location by an IoU of greater than 0.2. We decided to use the IoU cutoff of 0.2 because FLLs in USG images often have indistinct boundary, especially for FFSs and FFIs. **S2 Fig** demonstrated examples of detected FLLs with different IoUs.

S3 Appendix. Selection of confidence threshold

After image allocation into training and internal test set, a subset of images (5892 of 40397 images, 14.6%), i.e. tuning set, were randomly separated from the training set. Results of predictions by CNN on the tuning set were used to guide selection of an appropriate confidence threshold. For liver cancer surveillance, it is reasonable to emphasize on detection recall. We therefore utilized F2 score, which weighs recall higher than precision, to select the confidence threshold [2].

$$F2\ score = (1 + 2^2) \times \frac{recall \times precision}{recall + (2^2 \times precision)}$$

The appropriate confidence threshold was selected by analyzing plots between recalls, precisions and F2 scores versus confidence thresholds (**S8 Fig**). When the confidence threshold was decreased, recall increased, while precision decreased. The F2 scores were maximized and steady when the confidence thresholds ranged between 0.2 and 0.4. Confidence threshold of 0.2 was selected because it also yielded high recall, which was reasonable for liver cancer surveillance. The confidence threshold of 0.2 was then applied for evaluating AI system performance on internal test set and external validation dataset.

S4 Appendix. Proof of independence between confidence threshold and classification performance

Because the diagnosis performance could only be evaluated on the detected lesions (i.e. the lesions detected by predicted bounding boxes), and the number of detected lesions varies by different confidence thresholds (e.g. lowering confidence thresholds enables AI to detect more difficult-to-detect lesions), we hypothesized that the diagnosis performance on “difficult-to-detect” lesions may be different from that on “easy-to-detect” lesions. We tested this hypothesis by plotting confidence thresholds versus detection rates, diagnostic sensitivities and specificities (**S9 Fig**). From the plot, when confidence thresholds increased, detection rates decreased. Diagnostic sensitivities and specificities were almost constant when score thresholds were in the range of 0 to 0.5. Relating with score thresholds 0.2 – 0.4 that optimized F2 score, we concluded that at score thresholds at which the AI system best operated, the diagnostic sensitivities and specificities were comparable between “easy-to-detect” and “difficult-to-detect” lesions.

S1 Fig. Intersection-over-Union (IoU). White bounding boxes in the following example images denote true location, while orange bounding boxes denote predicted location.

Image	IoU
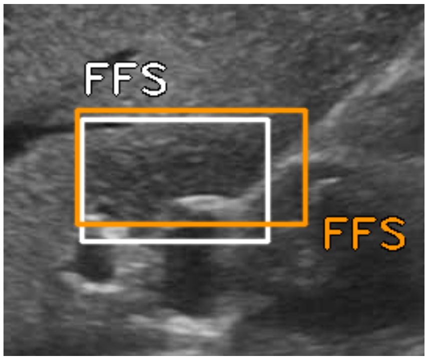	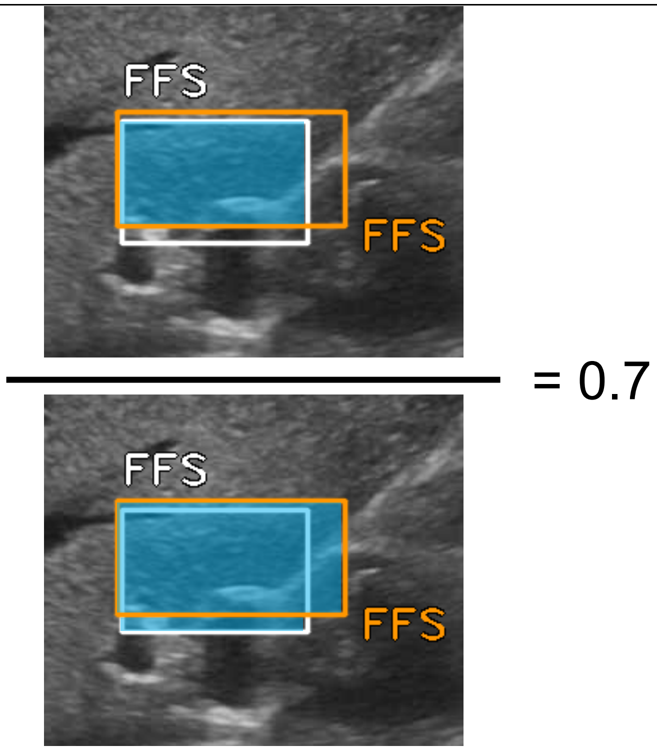

S2 Fig. Examples of detected FLLs with different IoUs. Images of FFSs are shown here because it is sometimes difficult to demarcate their exact boundary, hence justifying the use of IoU cutoff of 0.2. Images on the left panels are original images, images on the right panels are images with overlaid labeled (white) and AI-predicted (orange) bounding boxes.

**IoU**	**Original images**	**Images with labeled (white) and AI-predicted (orange) bounding boxes**
IoU = 0.26	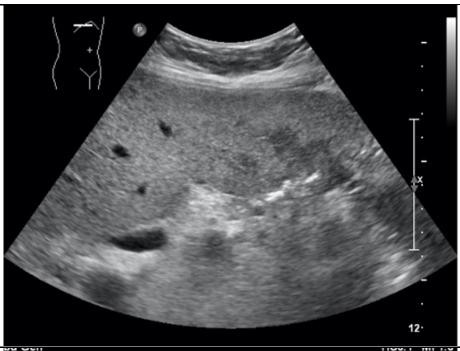	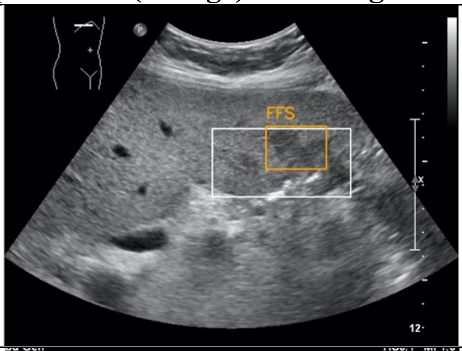
IoU = 0.38	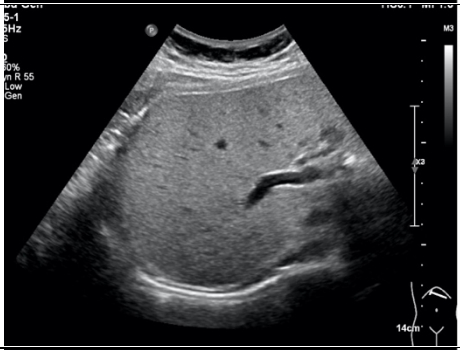	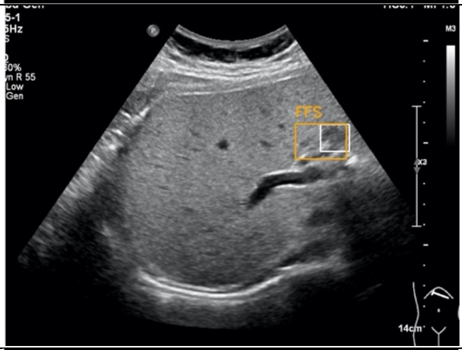
IoU = 0.54	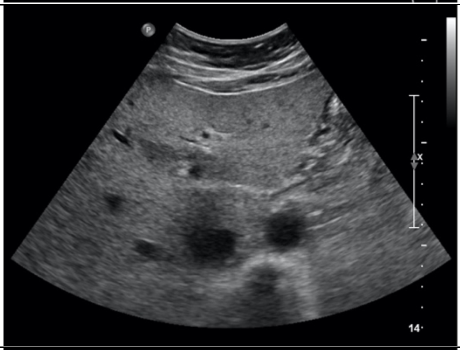	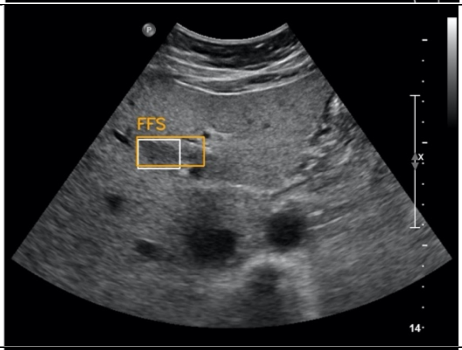
IoU = 0.91	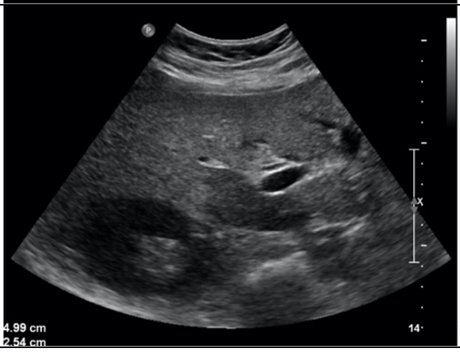	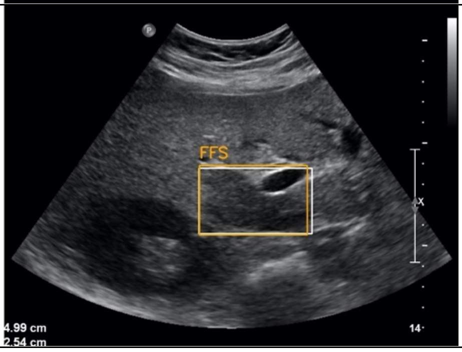

S3 Fig. Histogram shows distribution of IoUs of detected FLLs in the internal test set and the external validation set.

Distribution of IoUs of detected FLLs in the internal test set

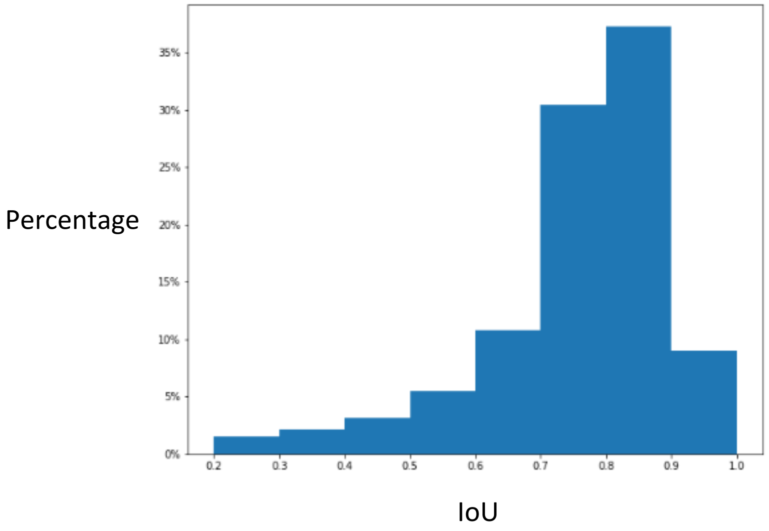

Distribution of IoUs of detected FLLs in the external validation dataset

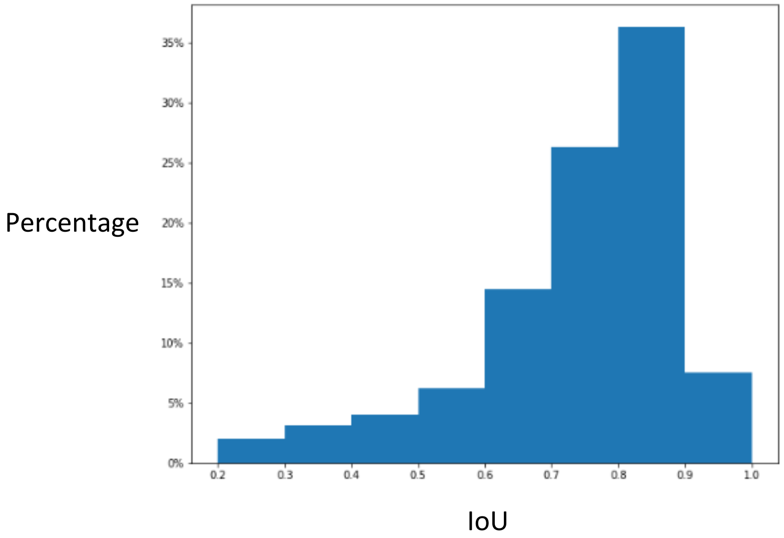

S4 Fig. Examples of normal organs and lesions in other organs falsely detected as FLLs. Colored bounding boxes indicate false positive predictions by the AI system.

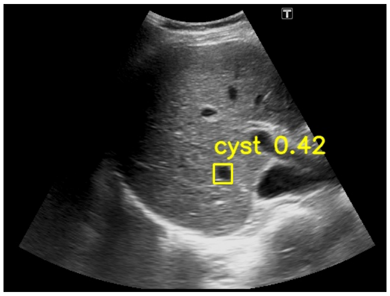

Blood vessel

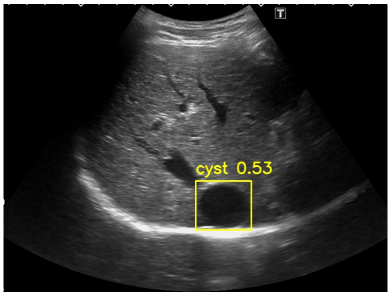

IVC

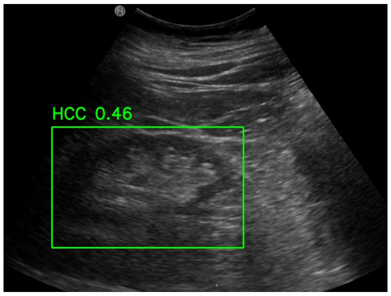

Kidney

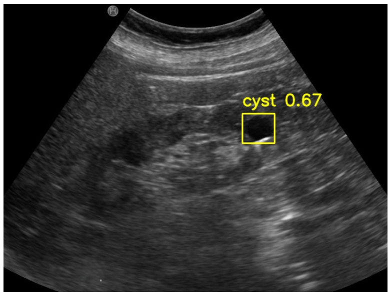

Renal cyst

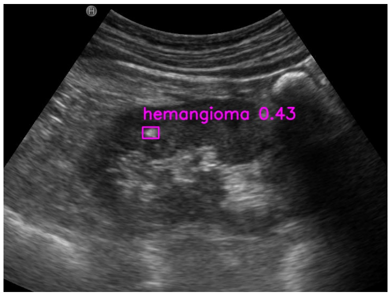

Renal stone

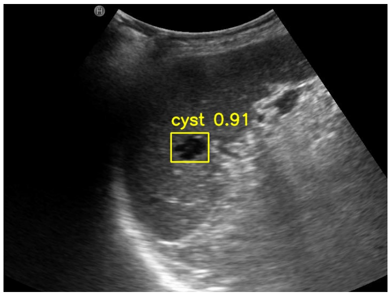

Splenic cyst

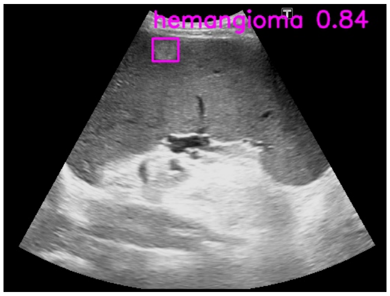

Splenic nodule

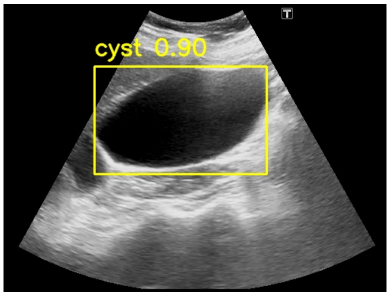

Gallbladder

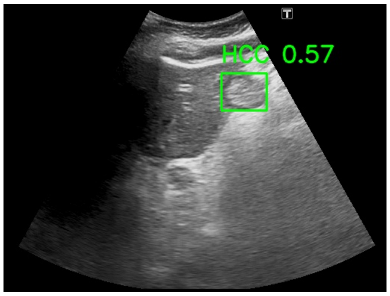

Stomach

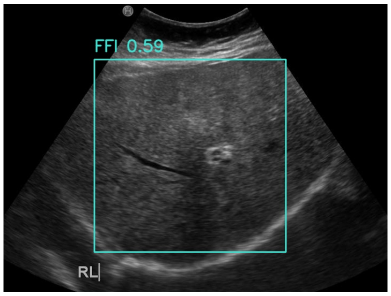

Heterogeneous liver background

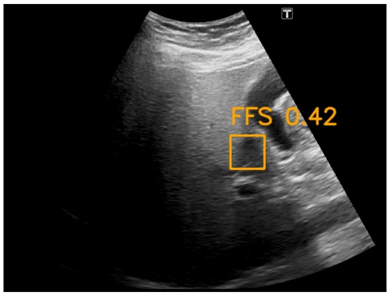

Shadow artifact

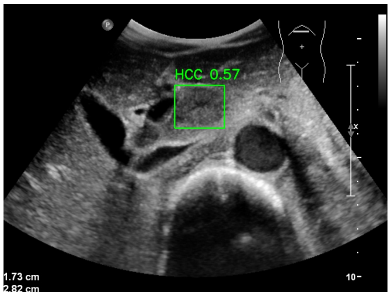

Enlarged lymph node

S5 Fig. Examples of false-negatively detected images. White bounding boxes indicate true location and definitive diagnosis.

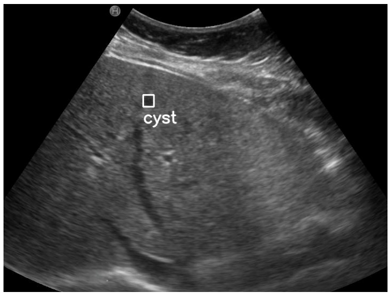

Small lesion (<1 cm)

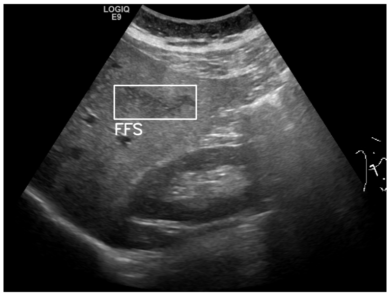

Uncommon location for FFS

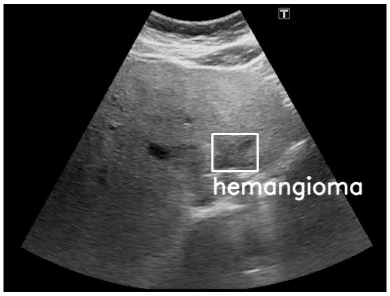

Atypical ill-defined hypoechoic hemangioma

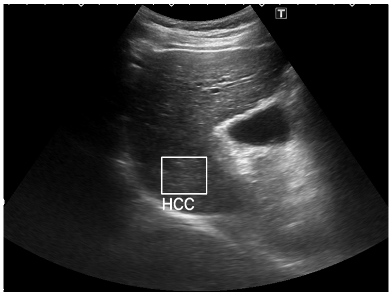

Ill-defined lesion

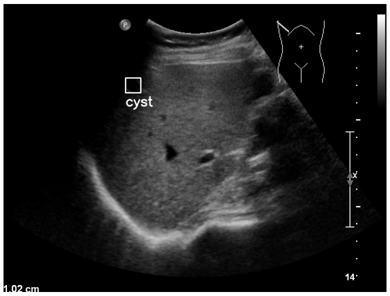

Lesion obscured by shadow artifacts

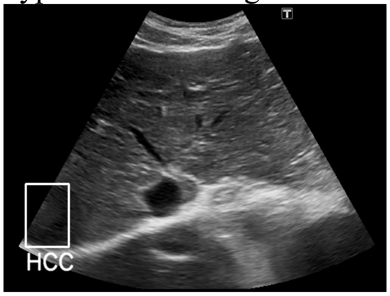

Lesion not completely seen

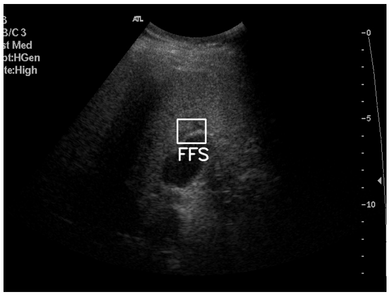

Dark or poor-quality image

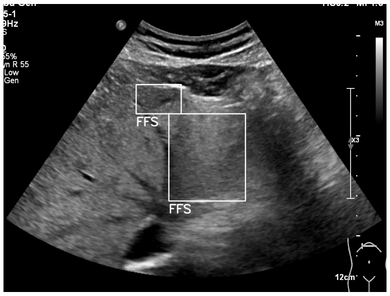

Heterogeneous liver background

S6 Fig. Examples of misclassified FLLs. White bounding boxes denote definitive diagnosis. Colored bounding boxes denote falsely predicted diagnosis by the AI system.

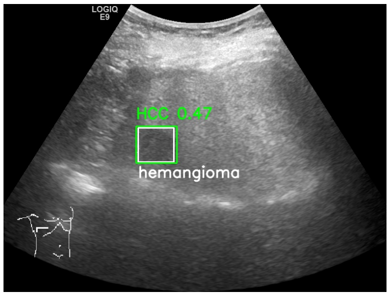

Hypoechoic hemangioma in fatty liver background; misclassified as HCC

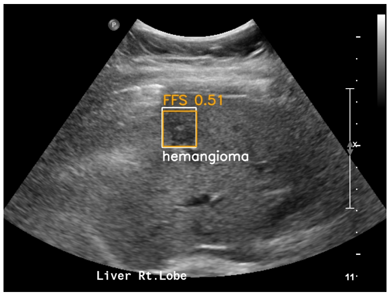

Hypoechoic hemangioma in fatty liver background; misclassified as FFS

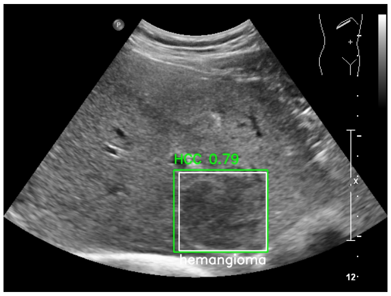

Giant heterogeneous hemangioma; misclassified as HCC

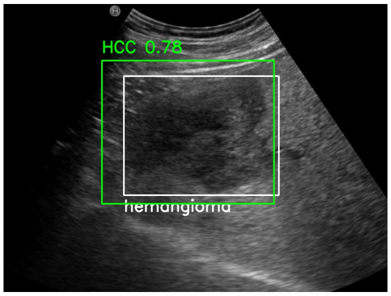

Giant heterogeneous hemangioma; misclassified as HCC

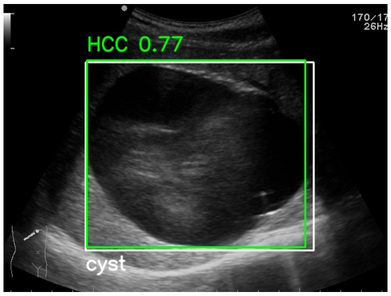

Complicated cyst; misclassified as HCC

S7 Fig. Examples of images before and after markers were removed

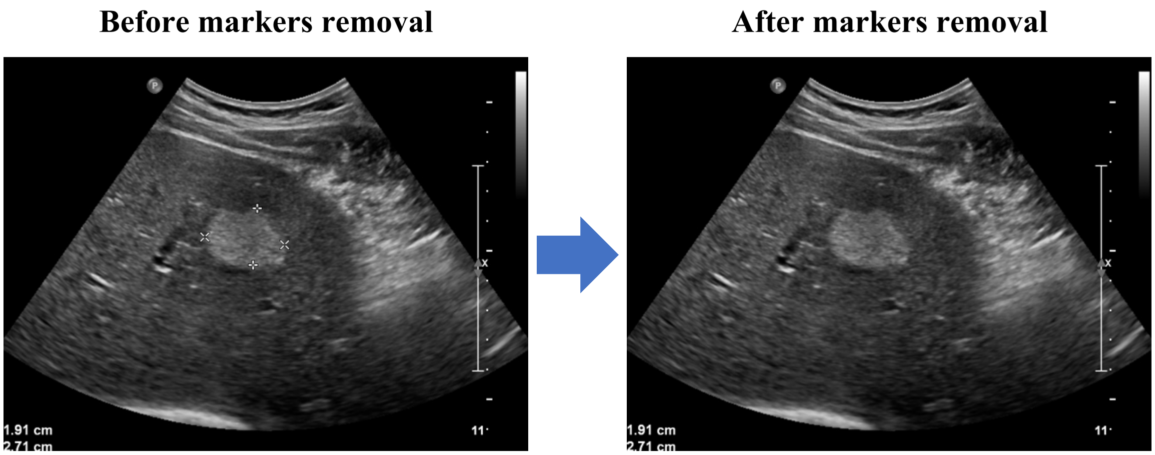

S8 Fig. Plots between score thresholds versus recalls, precisions and F2 scores for detecting each of the 5 FLL diagnoses. These plots were generated when the CNN was evaluated on a tuning set (5892 of 40397 images, 14.6%) that were randomly separated from the training set.

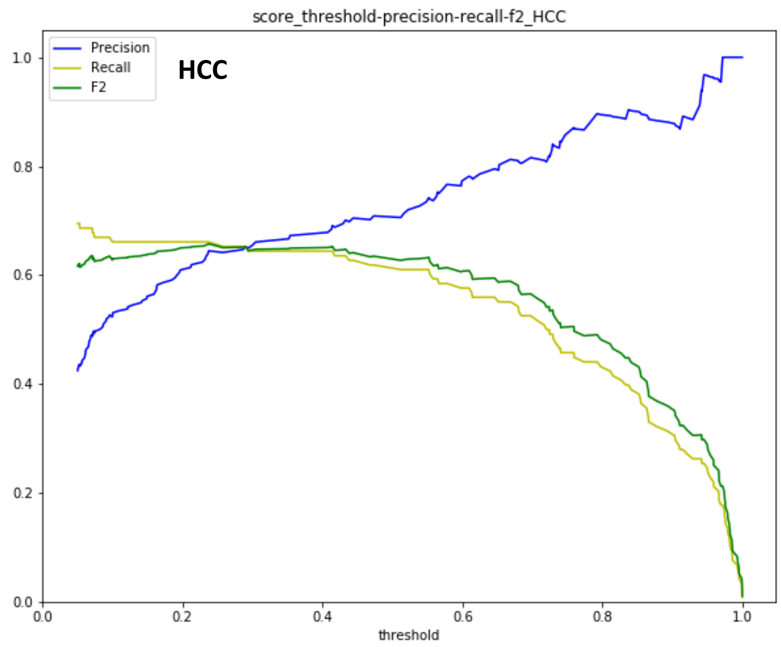

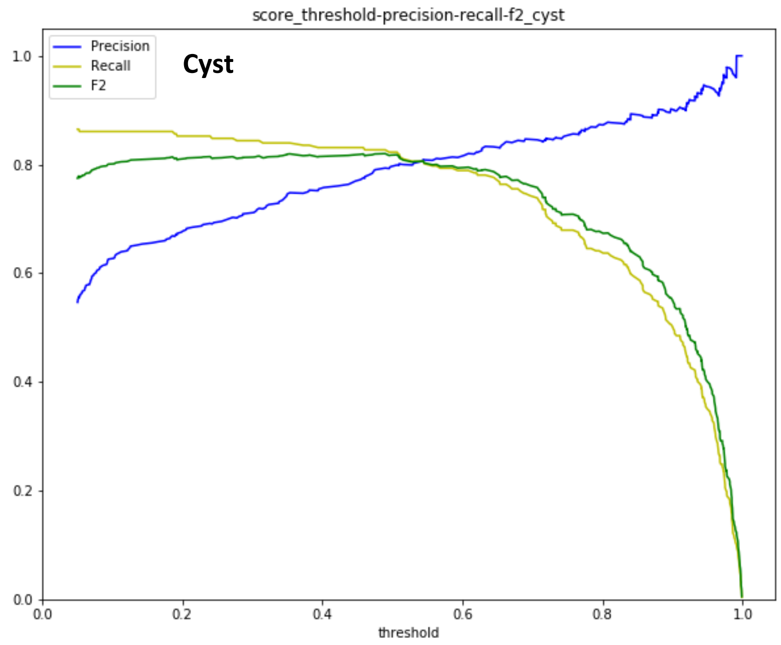

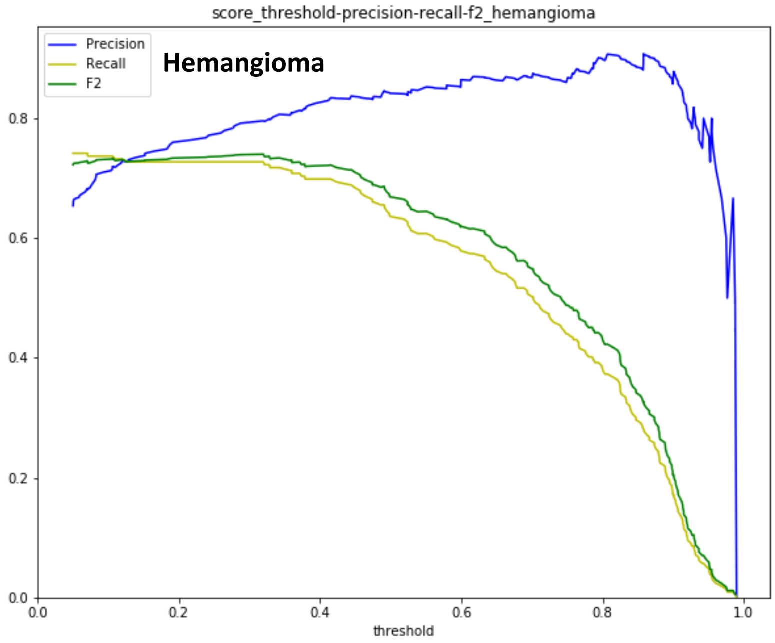
score_threshold-precision-recall-f2_hemangioma
Precision
Recall
F2
Hemangioma
0.0
0.2
0.4
0.6
0.8
1.0
threshold

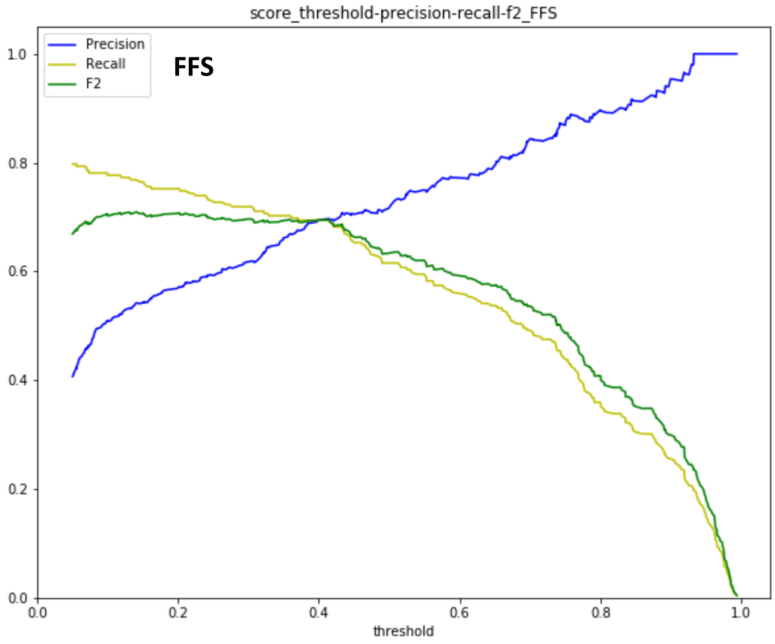
score_threshold-precision-recall-f2_FFS
Precision
Recall
F2
FFS
0.0
0.2
0.4
0.6
0.8
1.0
threshold

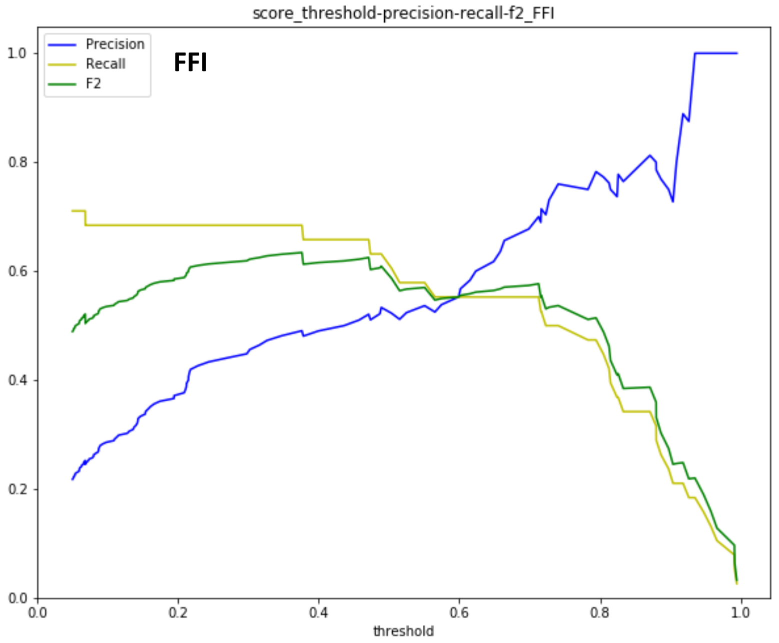
score_threshold-precision-recall-f2_FFI
Precision
Recall
F2
FFI
1.0
0.8
0.6
0.4
0.2
0.0
0.0
0.2
0.4
0.6
0.8
1.0
threshold

S9 Fig. Plots between score thresholds versus overall detection rates, diagnostic sensitivities and specificities for the internal test set and the external validation set

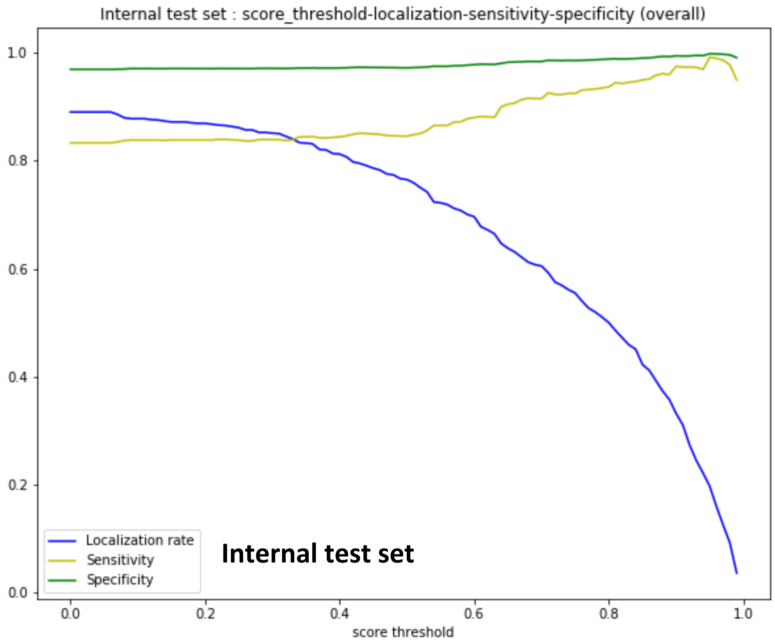

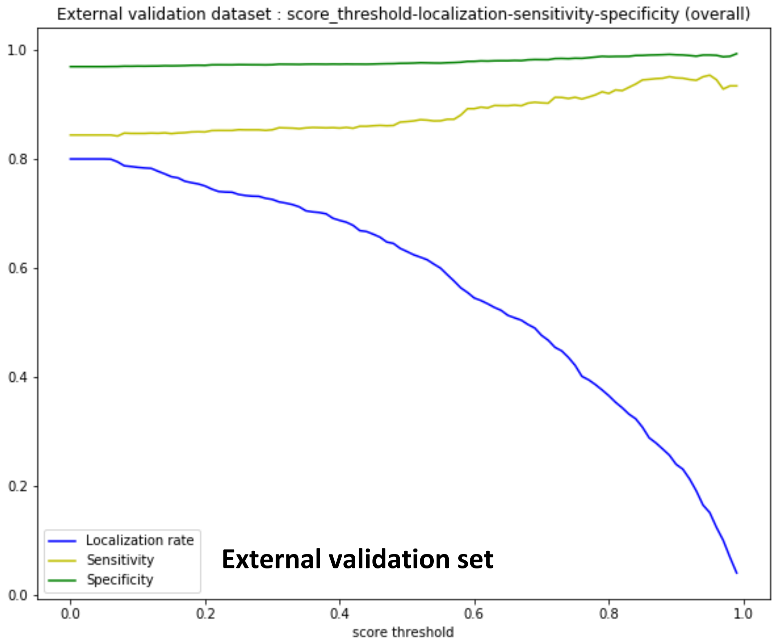

S1 Table. Ultrasound machine models from which images were taken. Percentages are shown in brackets.

Ultrasound machine models	Training set[a]	Internal test set[a]	External validation cohorts		
			Cohort 1[b]	Cohort 2[c]	Pooled
Total images	40397 (100)	6191 (100)	5624 (100)	13298 (100)	18922 (100)
ACUSON SEQUOIA	466 (1.2)	114 (1.8)	-	-	-
ALOKA CO. LTD. SSD-ALPHA6	-	-	521 (9.3)	-	521 (2.8)
ATL HDI 5000	375 (0.9)	22 (0.4)	-	-	-
GE Healthcare LOGIQE10	23 (0.1)	-	-	11 (0.1)	11 (0.1)
GE Healthcare LOGIQE9	6624 (16.4)	853 (13.8)	1903 (33.8)	5045 (37.9)	6948 (36.7)
GE Healthcare LOGIQF	-	-	246 (4.4)	-	246 (1.3)
GE Medical Systems LOGIQ9	1141 (2.8)	112 (1.8)	-	-	-
HITACHIMEDICAL CORPORATION EUB-7000HV	-	-	15 (0.3)	-	15 (0.1)
Hitachi Medical Corporation HI VISION Avius	-	-	2939 (52.3)	-	2939 (15.5)
Philips Medical Systems EPIQ 5C	64 (0.2)	12 (0.2)	-	-	-
Philips Medical Systems EPIQ 7G	3137 (7.8)	845 (13.6)	-	84 (0.6)	84 (0.4)
Philips Medical Systems iU22	28021 (69.4)	4192 (67.7)	-	-	-
SAMSUNG MEDISON CO. LTD. RS80A	14 (0.0)	-	-	7 (0.1)	7 (0.0)
SuperSonic Imagine SA Aixplorer	73 (0.2)	-	-	-	-
SuperSonic Imagine SA AixplorerUltimate	-	-	-	10 (0.1)	10 (0.1)
TOSHIBA MEC AplioXG	-	-	-	5940 (44.7)	5940 (31.4)
TOSHIBA MEC US TUS-A500	40 (0.1)	16 (0.3)	-	2201 (16.6)	2201 (11.6)
others	419 (1.0)	25 (0.4)	-	-	-

[a]KCMH, King Chulalongkorn Memorial Hospital, Bangkok, Thailand

[b]Mahachai Hospital, Samut Sakhon, Thailand

[c]Queen Savang Vadhana Memorial Hospital, Chonburi, Thailand

S2 Table. False-positive detection of other structures as FLLs. Numbers shown are images. Percentages are shown in brackets.

Structures false-positively detected as FLLs	False-positively detected FLL diagnoses						
	**Total**	**HCC**	**cyst**	**hemangioma**	**FFS**	**FFI**	**>1 predictions**
blood vessel	147 (12.3) [a]	-	91 (24.7) [a]	12 (9.9) [b]	21 (11.6) [a]	-	23 (14.8) [a]
portal vein	2 (0.2)	-	2 (0.5)	-	-	-	-
IVC	41 (3.4) [d]	-	33 (8.9) [c]	-	3 (1.7) [d]	-	5 (3.2) [b]
aorta	1 (0.1)	-	1 (0.3)	-	-	-	-
CBD	2 (0.2)	-	1 (0.3)	-	1 (0.6)	-	-
kidney	19 (1.6)	11 (5.4) [b]	-	3 (2.5)	2 (1.1) [e]	1 (0.6) [e]	2 (1.3) [e]
renal cyst	81 (6.8) [c]	7 (3.4) [c]	72 (19.5) [b]	-	-	-	2 (1.3) [e]
renal stone	13 (1.1)	-	-	10 (8.3) [c]	-	1 (0.6) [e]	2 (1.3) [e]
renal AML	3 (0.3)	-	-	3 (2.5)	-	-	-
unspecified renal lesions	8 (0.7)	3 (1.5) [e]	-	4 (3.3)	-	-	1 (0.6)
spleen	4 (0.3)	1 (0.5)	1 (0.3)	2 (1.7)	-	-	-
splenic cyst	10 (0.8)	1 (0.5)	8 (2.2) [e]	1 (0.8)	-	-	-
unspecified splenic lesions	33 (2.8) [e]	6 (2.9) [d]	1 (0.3)	21 (17.4) [a]	1 (0.6)	1 (0.6) [e]	3 (1.9) [d]
gallbladder	31 (2.6)	-	25 (6.8) [d]	-	2 (1.1) [e]	-	4 (2.6) [c]
gallbladder polyp	2 (0.2)	-	-	2 (1.7)	-	-	-
gallstone	1 (0.1)	-	-	-	-	-	1 (0.6)
gallbladder wall	1 (0.1)	-	-	-	-	1 (0.6) [e]	-
pancreas	5 (0.4)	1 (0.5)	1 (0.3)	1 (0.8)	-	2 (1.2) [d]	-
pancreatic solid mass	3 (0.3)	3 (1.5) [e]	-	-	-	-	-
pancreatic cyst	2 (0.2)	-	2 (0.5)	-	-	-	-
stomach	6 (0.5)	6 (2.9) [d]	-	-	-	-	-
heart	1 (0.1)	-	1 (0.3)	-	-	-	-
abdominal organs*	7 (0.6)	2 (1.0)	3 (0.8)	1 (0.8)	-	-	1 (0.6)
perivascular soft tissue	17 (1.4)	-	-	4 (3.3)	-	8 (4.8) [b]	5 (3.2) [b]
peri-gallbladder soft tissue	2 (0.2)	-	-	1 (0.8)	-	1 (0.6) [e]	-
heterogeneous liver background	88 (7.4) [b]	34 (16.6) [a]	-	9 (7.4) [d]	9 (5.0) [c]	33 (20.0) [a]	3 (1.9) [d]
artifacts	31 (2.6)	2 (1.0)	2 (0.5)	7 (5.8) [e]	13 (7.2) [b]	3 (1.8) [c]	4 (2.6) [c]
pseudolesion	12 (1.0)	-	-	7 (5.8) [e]	-	2 (1.2) [d]	3 (1.9) [d]
cirrhotic nodule	4 (0.3)	-	-	4 (3.3)	-	-	-
ascites	2 (0.2)	-	2 (0.5)	-	-	-	-
enlarged lymph node	2 (0.2)	1 (0.5)	-	-	1 (0.6)	-	-
miscellaneous	615 (51.4)	127 (62.0)	123 (33.3)	29 (24.0)	128 (70.7)	112 (67.9)	96 (61.9)
Total	1196 (100)	205 (100)	369 (100)	121 (100)	181 (100)	165 (100)	155 (100)

* abdominal organs other than kidney, spleen, gallbladder, pancreas, stomach and heart

[a, b, c, d, e] For each diagnosis false-positively detected, ‘a’, ‘b’, ‘c’, ‘d’, ‘e’ denotes the first, second, third, fourth and fifth most common structures that were false-positively detected as the FLL diagnosis.

S3 Table. Causes of false negative detections

**Causes**	**N (image)**	**%**
small lesions (<1 cm)	106	27.4
uncommon location for particular diagnosis	31	8.0
atypical characteristics	30	7.8
ill-defined lesions	29	7.5
lesion obscured by shadow artifacts or not completely seen	24	6.2
dark or poor-quality images	18	4.7
heterogeneous liver background	3	0.8
miscellaneous	146	37.7
TOTAL	387	100

S4 Table. Causes of misclassifications. Numbers shown are images. Percentages are shown in brackets.

**Causes of misclassifications**	**definitive diagnoses**					
	**Total**	**HCC**	**cyst**	**hemangioma**	**FFS**	**FFI**
atypical characteristics*	56 (30.2)	-	2 (14.3)	54 (53.5)	-	-
hypoechoic	34	NA	NA	34	NA	NA
heterogeneous	20	NA	NA	20	NA	NA
complicated cyst	2	NA	2	NA	NA	NA
small lesions (<1 cm)	7 (3.8)	6 (14.0)	-	1 (1.0)	-	-
uncommon location for particular diagnosis	3 (1.6)	-	-	-	-	3 (15.0)
lesion obscured by shadow artifacts or not completely seen	2 (1.1)	2 (4.7)	-	-	-	-
ill-defined lesions	1 (0.5)	-	-	-	-	1 (5.0)
dark or poor-quality images	1 (0.5)	-	-	-	1 (12.5)	-
miscellaneous	116 (62.4)	35 (81.4)	12 (85.7)	46 (45.5)	7 (87.5)	16 (80.0)
Total	186 (100)	43 (100)	14 (100)	101 (100)	8 (100)	20 (100)

* For an FLL diagnosis, only some atypical characteristics are applicable.

S5 Table. Subgroup analysis by FLL sizes. 95% CIs are given in parentheses.

	Internal test set			External validation set		
	< 2 cm	2 - 3 cm	> 3 cm	< 2 cm	2 - 3 cm	> 3 cm
**Overall**						
N	532	140	173	696	243	256
Detection rate	91.4 (87.9-94.9)	84.6 (78.1-91.2)	79.6 (73.1-86.0)	76.4 (71.4-81.3)	75.3 (67.9-82.6)	78.1 (72.4-83.8)
Diagnostic sensitivity	71.4 (65.1-77.8)	85.0 (77.9-92.1)	85.9 (79.6-92.2)	81.8 (76.3-87.3)	85.1 (78.0-92.1)	76.6 (69.1-84.2)
Diagnostic specificity	97.3 (96.6-98.0)	96.3 (94.6-98.0)	97.0 (95.6-98.4)	97.7 (97.1-98.4)	97.2 (95.9-98.5)	94.5 (92.9-96.1)
**HCC**						
N	19	25	58	31	24	83
Detection rate	89.5 (75.7-100)	88.0 (75.3-100)	82.8 (73.0-92.5)	77.4 (62.7-92.1)	79.2 (62.9-95.4)	78.3 (69.4-87.2)
Diagnostic sensitivity	23.5 (3.4-43.7)	77.3 (59.8-94.8)	89.6 (80.9-98.2)	50.0 (30.0-70.0)	84.2 (67.8-100)	92.3 (85.8-98.8)
Diagnostic specificity	98.5 (97.4-99.6)	95.7 (91.7-99.8)	96.6 (92.7-100)	97.8 (96.5-99.1)	94.3 (90.7-97.9)	82.1 (75.6-88.6)
**Cyst**						
N	178	15	22	181	22	14
Detection rate	91.0 (86.8-95.2)	80.0 (59.8-100)	81.8 (65.7-97.9)	77.9 (71.9-83.9)	90.9 (78.9-100)	100 (76.8-100)
Diagnostic sensitivity	98.1 (96.1-100)	100 (73.5-100)	94.4 (83.9-100)	95.7 (92.4-99.1)	90.0 (76.9-100)	85.7 (67.4-100)
Diagnostic specificity	98.4 (97.0-99.8)	98.1 (95.4-100)	98.3 (95.9-100)	98.4 (97.2-99.7)	98.7 (97.0-100)	98.4 (96.6-100)
**Hemangioma**						
N	163	31	23	160	48	54
Detection rate	93.9 (90.2-97.5)	100 (88.8-100)	82.6 (67.1-98.1)	82.5 (76.6-88.4)	68.8 (55.6-81.9)	79.6 (68.9-90.4)
Diagnostic sensitivity	85.0 (79.3-90.6)	67.7 (51.3-84.2)	68.4 (47.5-89.3)	80.3 (73.5-87.1)	63.6 (47.2-80.0)	39.5 (24.9-54.1)
Diagnostic specificity	95.1 (92.7-97.4)	95.3 (90.8-99.8)	94.8 (90.8-98.9)	96.9 (95.2-98.6)	99.3 (97.9-100)	94.2 (90.6-97.9)
**FFS**						
N	155	57	52	300	137	87
Detection rate	82.6 (76.6-88.6)	71.9 (60.3-83.6)	67.3 (54.6-80.1)	69.0 (63.8-74.2)	70.8 (63.2-78.4)	77.0 (68.2-85.9)
Diagnostic sensitivity	97.7 (95.0-100)	100 (91.4-100)	97.1 (91.6-100)	99.5 (98.6-100)	100 (96.3-100)	95.5 (90.6-100)
Diagnostic specificity	97.1 (95.4-98.9)	96.0 (91.6-100)	97.0 (93.7-100)	98.1 (96.6-99.6)	95.0 (90.2-99.8)	97.7 (95.2-100)
**FFI**						
N	17	12	18	24	12	18
Detection rate	100 (80.5-100)	83.3 (62.2-100)	83.3 (66.1-100)	75.0 (57.7-92.3)	66.7 (40.0-93.3)	55.6 (32.6-78.5)
Diagnostic sensitivity	52.9 (29.2-76.7)	80.0 (55.2-100)	80.0 (59.8-100)	83.3 (66.1-100)	87.5 (64.6-100)	70.0 (41.6-98.4)
Diagnostic specificity	97.4 (95.9-98.8)	96.2 (92.6-99.9)	98.3 (96.0-100)	97.4 (96.0-98.8)	98.8 (97.2-100)	100 (98.1-100)

S6 Table. Subgroup analysis by background liver parenchyma. 95% CIs are given in parentheses.

	Internal test set		External validation set	
	**Cirrhosis**	**Non-cirrhosis**	**Cirrhosis**	**Non-cirrhosis**
**Overall**				
N	72	773	142	1053
Detection rate	79.9 (65.9-93.9)	88.0 (85.2-90.8)	48.8 (35.0-62.5)	78.7 (75.4-82.1)
Diagnostic sensitivity	84.3 (70.5-98.2)	83.9 (79.8-87.9)	NA	84.7 (80.7-88.6)
Diagnostic specificity	94.5 (91.4-97.7)	97.1 (96.5-97.7)	95.0 (92.3-97.8)	97.3 (96.7-97.8)
**HCC**				
N	44	58	100	38
Detection rate	79.5 (67.6-91.5)	89.7 (81.8-97.5)	72.0 (63.2-80.8)	94.7 (87.6-100)
Diagnostic sensitivity	80.0 (66.7-93.3)	69.2 (56.7-81.8)	81.9 (73.1-90.8)	80.6 (67.6-93.5)
Diagnostic specificity	90.9 (78.9-100)	98.1 (97.0-99.1)	89.3 (77.8-100)	94.6 (93.0-96.2)
**Cyst**				
N	5	210	24	193
Detection rate	80.0 (44.9-100)	89.5 (85.4-93.7)	83.3 (68.4-98.2)	79.8 (74.1-85.5)
Diagnostic sensitivity	100 (39.8-100)	97.9 (95.8-99.9)	95.0 (85.4-100)	94.2 (90.5-97.9)
Diagnostic specificity	94.3 (88.1-100)	98.8 (97.8-99.7)	97.5 (94.1-100)	98.6 (97.7-99.5)
**Hemangioma**				
N	15	202	10	252
Detection rate	80.0 (59.8-100)	94.6 (91.4-97.7)	60.0 (29.6-90.4)	80.2 (75.2-85.1)
Diagnostic sensitivity	75.0 (50.5-99.5)	81.2 (75.6-86.7)	66.7 (28.9-100)	69.3 (62.9-75.7)
Diagnostic specificity	91.1 (82.8-99.4)	95.4 (93.5-97.3)	90.4 (84.5-96.4)	97.8 (96.6-99.0)
**FFS**				
N	5	259	7	517
Detection rate	60.0 (17.1-100)	77.6 (72.5-82.7)	28.6 (0-62.0)	71.0 (67.1-74.9)
Diagnostic sensitivity	100 (29.2-100)	98.0 (96.1-99.9)	100 (15.8-100)	98.9 (97.8-100)
Diagnostic specificity	100 (93.4-100)	96.6 (95.0-98.2)	99.0 (97.0-100)	97.2 (95.6-98.8)
**FFI**				
N	3	44	1	53
Detection rate	100 (29.2-100)	88.6 (79.3-98.0)	0 (0-97.5)	67.9 (55.4-80.5)
Diagnostic sensitivity	66.7 (13.3-100)	69.2 (54.7-83.7)	NA	80.6 (67.6-93.5)
Diagnostic specificity	96.3 (91.3-100)	97.5 (96.2-98.7)	99.0 (97.0-100)	98.2 (97.2-99.1)

NA, not applicable

S7 Table. top-1, top-2 and top-3 diagnostic sensitivities and specificities for HCC. 95% CIs are given in parentheses.

	**Internal test set**	**External validation set**
**Diagnostic sensitivity for HCC**		
Top-1	73.6 (64.3 – 82.8)	81.5 (74.2 – 88.8)
Top-2	90.8 (84.7 – 96.9)	89.0 (83.1 – 94.9)
Top-3	96.6 (92.7 – 100)	93.6 (89.0 – 98.2)
**Diagnostic specificity for HCC**		
Top-1	97.8 (96.7 – 98.9)	94.4 (92.8 – 96.0)
Top-2	85.5 (82.8 – 88.3)	83.6 (81.0 – 86.2)
Top-3	78.5 (75.4 – 81.7)	78.9 (76.0 – 81.7)

S8 Table. Training hyperparameters for RetinaNet

**Training hyperparameters**	**Values**
Iterations (= [epochs] x [steps per epoch])	500,000 (25 epochs × 20,000 steps per epoch)
Batch size	2
Input size (width, height)	(1333, 800)
Image mean (grayscale)	46.15
Initial learning rate	0.0001
Learning rate scheduling	Decrease learning rate by a factor of 0.5 if there is no decrease in validation loss for 2 epochs
Anchor sizes	[32, 64, 128, 256, 512]
Anchor strides	[8, 16, 32, 64, 128]
Anchor ratios	[0.5, 1, 2]
Anchor scales	[1, $2^{1/3}$, $2^{2/3}$]
IoU for Negative overlap	< 0.2
IoU for Positive overlap	> 0.5
**Image augmentation**	
- Rotation	± 0.1 radian
- Translation factor	± 0.1 in x and y direction
- Scaling factor	± 0.1
- Horizontal flip probability	0.2
- Contrast range	(0.9, 1.1)
- Brightness range	(0.9, 1.1)
- Hue parameter range	(-0.05, 0.05)
- Saturation range	(0.95, 1.05)
- Motion blur	Motion in x-direction (horizontal)

References

1. Radford A, Metz L, Chintala S. Unsupervised representation learning with DCGAN. arXiv preprint arXiv:151106434. 2015.

2. Sasaki Y. The truth of the F-measure. Teach Tutor mater. 2007;1(5):1-5.
